# Comparison of Six-minute Walk Test Parameters by Severity of Idiopathic and Non-idiopathic Pulmonary Fibrosis

**DOI:** 10.1298/ptr.E10288

**Published:** 2025-06-25

**Authors:** Masashi ZENTA, Kenji TSUSHIMA, Tomohiro HATTORI, Jin KUBO, Natsuko MIYAMAE, Minami AKAMA, Satoshi KIDO

**Affiliations:** 1 Division of Rehabilitation, International University of Health and Welfare (IUHW) Ichikawa Hospital, Japan; 2 Department of Health and Social Services, Course of Health and Social Services, Graduate School of Saitama Prefectural University, Japan; 3 Department of Pulmonary Medicine, Tokyo Medical University Hachioji Medical Center, Japan; 4 Department of Pulmonary Medicine, International University of Health and Welfare (IUHW) Ichikawa Hospital, Japan; 5 Department of Rehabilitation Medicine, Southern-Tohoku General Hospital, Japan

**Keywords:** 6-minute walk test, Idiopathic pulmonary fibrosis, Interstitial lung disease, Severity, GAP stage

## Abstract

Objectives: The 6-minute walk test (6MWT) is generally used to evaluate endurance in interstitial lung disease (ILD). In ILD, treatment efficacy differs between patients with idiopathic pulmonary fibrosis (IPF) and those with non-idiopathic pulmonary fibrosis (non-IPF), and the clinical profile varies according to disease severity. This study compared 6MWT parameters by severity of illness in patients with IPF and non-IPF. Methods: The participants were hospitalized patients with ILD and either IPF (n = 20) or non-IPF (n = 25). To compare IPF and non-IPF by severity, patients were classified using the gender–age–physiology (GAP) index as having GAP stage I (i.e., mild) or GAP stage II/III (i.e., moderate to severe). The evaluation parameters were the 6-minute walk distance (6MWD), minimum percutaneous oxygen saturation (SpO_2_), heart rate recovery at 1 minute (HRR1) after the 6MWT, and modified Borg scale rating of perceived exertion. Results: The minimum SpO_2_ value during exertion was significantly lower in the IPF group than in the non-IPF group (p < 0.05). Furthermore, when comparing by severity, patients with IPF and GAP stage I had a significantly lower 6MWD, HRR1, and SpO_2_ value. In patients with GAP stage II/III, there were no significant differences in 6MWD, SpO_2_ values, or dyspnea. Conclusions: Patients with mild IPF were more prone to hypoxemia, while in moderate-to-severe cases, we observed no significant hypoxemia- or endurance-related differences between IPF and non-IPF patients. This study highlights the importance of severity-based evaluation, particularly in guiding individualized rehabilitation and risk management for patients with IPF.

## Introduction

Interstitial lung disease (ILD) is a general term for diseases characterized by inflammation and fibrosis in the interstitium of the lungs; it is estimated that more than 200 types of diseases can cause ILD^1)^. In ILD, the prognosis and treatment efficacy differ between the presence of idiopathic pulmonary fibrosis (IPF) and non-idiopathic pulmonary fibrosis (non-IPF)^2)^. Although patients with IPF have a poorer prognosis than those with non-IPF forms^3,4)^, both groups have exhibited even worse outcomes when progressive pulmonary fibrosis (PPF) is present in recent years^5)^.

The 6-minute walk test (6MWT) is widely used for endurance assessment and prognosis in patients with ILD^6)^. Previous comparative studies applying the 6MWT in IPF and non-IPF patients have described inconsistent results concerning hypoxemia, with one study indicating no hypoxemia-related difference between the groups^7)^, and another reporting significantly higher hypoxemia levels in patients with IPF^8)^. These studies did not stratify patients by illness severity using the gender–age–physiology (GAP) stage, which may have contributed to the inconsistent results.

Similarly, the GAP stage reportedly also displays a prognostic value. The GAP model, which uses gender, age, and lung function for staging, has been reported as a prognostic model for ILD^9)^. The GAP stage is associated with both forced vital capacity (FVC) and diffusion capacity of the lung (DLco), with more severe disease stages correlating with a higher likelihood of hypoxemia^9)^. Compared with GAP stage I IPF patients, GAP stage II/III IPF patients had a significantly shorter exacerbation period and poorer prognosis^10)^. Other studies have reported a lower quality of life^11)^ and increased mortality^12)^ in GAP stages II and III compared to those in GAP stage I. In addition, they had lower 6-minute walk distance (6MWD) and lower minimum SpO_2_ during the 6MWT. Thus, evaluation by GAP stage is important to understand endurance weakness and exertion-induced hypoxemia. However, the 6MWT by GAP stage has only been reported in IPF patients and has not been validated in non-IPF patients.

We hypothesized that some patients with moderate-to-severe non-IPF may have a prognosis and exhibit trends such as hypoxemia and decreased endurance that are similar to those of patients with IPF. Understanding these differences may be significant for predicting hypoxemia and endurance loss during the 6MWT, potentially aiding in risk management. Therefore, the purpose of this study was to compare 6MWT parameters between IPF and non-IPF patients by disease severity and to examine the differences in these parameters.

## Methods

### Study design, patients, and setting

This retrospective cross-sectional study involved 105 patients hospitalized with ILD in the Pulmonary Medicine Ward of the International University of Health and Welfare, Ichikawa Hospital (Japan) from August 2017 to September 2019. All patients were eligible for pulmonary physiotherapy. Causes for hospitalization of participants were evaluation for diagnosis, medication adjustment, and educational hospitalization for oxygen induction and flow adjustment. Two patients with IPF (10%) and 1 with non-IPF (4%) were hospitalized for acute exacerbations and underwent the 6MWT at a median of 25 days (interquartile range 24–30 days) after their condition stabilized. In accordance with the methods used in a previous study^13)^, exclusion criteria were difficulty performing the 6MWT or difficulty understanding instructions, ventilator management, cardiac or cerebrovascular disease requiring treatment, and cancer. The diagnosis of ILD was determined by specialists in diffuse lung diseases and imaging physicians, based on the 2018 IPF treatment guidelines^14)^. IPF was diagnosed when the usual interstitial pneumonia pattern was identified on high-resolution computed tomography imaging. In challenging cases, bronchoalveolar lavage was performed and patients were classified as having IPF or non-IPF based on respiratory function, blood test data, and physical examination findings.

### Outcomes

1. 6MWT assessment

For the 6MWT method, a 30-m flat straight walking path was used with a turnaround, with measurements taken by 3 persons familiar with pulmonary rehabilitation for at least 5 years. In accordance with the guideline method^15)^, the participants did not walk unaccompanied during the test, and voice calls were made every minute as determined by the guideline. The 6MWT was conducted twice on the same day, with a minimum 15-minute rest interval between tests. Measurements included percutaneous oxygen saturation (SpO_2_), hear rate (HR), and the modified Borg scale every minute.

2. GAP stage-based severity classification

The GAP index was used to determine disease severity in IPF and non-IPF patients because it has been proposed as a superior prognostic severity classification for ILD patients^12)^. Gender, age, and lung function (i.e., FVC and DLco) are considered in the GAP index, which has a maximum score of 8. The total point score is used to classify patients by GAP stage as follows: GAP stage I (0–3 points), GAP stage II (4–5 points), or GAP stage III (6–8 points). In accordance with the findings of a previous study^11)^, patients were grouped into GAP stage I for mild disease and GAP stage II/III for moderate-to-severe disease.

3. Evaluation items for pulmonary function tests

Pulmonary function tests were conducted to measure vital capacity (VC), FVC, and DLco. Severe diffusion impairment was defined as a DLco of 45% or lower, based on a previous study^16)^.

4. PPF definition

According to a position paper^17)^, PPF is defined by the following criteria: (1) a 10% decrease in %FVC; (2) a decrease of 15% or more in %DLco; (3) a decline of 5% to 10% in %FVC, accompanied by subjective symptoms and fibrotic changes observed on imaging; and (4) exacerbation of imaging findings and subjective symptoms, irrespective of respiratory function.

5. Primary endpoint and other variables collected

The primary endpoint was the 6MWT parameters, which were measured within 1 week before discharge. The specific endpoints were the 6MWD, the minimum SpO_2_ during exertion, the modified Borg scale score at the end of the 6MWT on perceived exertion, and the maximum pulse rate. Heart rate recovery at 1 minute (HRR1) after the 6MWT was also determined by using the pulse rate at the end of the 6MWT minus the pulse rate 1 minute after the end of the 6MWT. Other evaluated endpoints included respiratory function tests, blood gas tests, echocardiography, biochemical tests, and knee extension strength within 1 week before discharge.

### Statistical analysis

Power analyses were performed using G^*^Power version 3.1.9.6 (Heinrich-Heine-Universität, Düsseldorf, Germany) for sample size calculations. Possible effect sizes were calculated based on SpO_2_ of IPF and non-IPF in the preliminary study. Assuming an effect size of 0.78, a significance level of 5%, and a power of 80%, the total sample size required was 44 cases. The Student's *t*-test was used for normally distributed continuous variables, the Mann–Whitney *U*-test for non-normally distributed continuous variables, and the chi-squared test for categorical variables. Two-way analysis of variance was used to compare groups based on disease severity, and the Bonferroni method was used for multiple comparison tests. For multiple comparisons, Bonferroni correction was applied to each subgroup analysis of patients with IPF vs. those with non-IPF within each GAP stage. The Steel–Dwass method was used for nonparametric multiple comparisons. SPSS version 18.0 (SPSS Inc., Chicago, IL, USA) or JMP version 12.2 (SAS Institute, Cary, NC, USA) was used to perform the analysis, with a significance level of 5%.

### Ethical considerations

This study was approved by the Ethical Review Committee of Health and Welfare Ichikawa Hospital (receipt number: 52) and Graduate School of Saitama Prefectural University (receipt number: 30506), and written informed consent was obtained from each participant.

## Results

### Participants’ characteristics

**Fig. 1.**
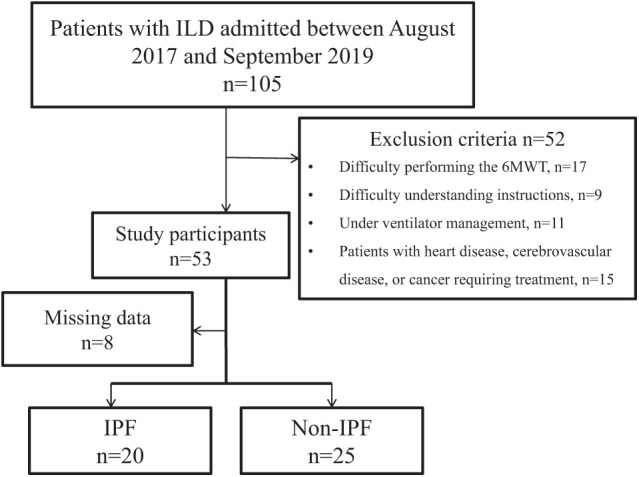
Flowchart of participants’ inclusion in the study ILD, interstitial lung disease; 6MWT, 6-minute walk test; IPF, idiopathic pulmonary fibrosis

Of the 105 hospitalized patients who received respiratory physiotherapy, 45 consecutive cases were finally included in the analysis, after excluding 52 patients who met the exclusion criteria and 8 patients with missing data. The majority of the 17 patients excluded from the 6MWT were unable to participate because of walking difficulties (11 patients, 65%). The participants were divided into 2 groups: IPF (n = 20) and non-IPF (n = 25) (Fig. 1). The patients were classified into mild (GAP stage I; IPF, n = 7; non-IPF, n = 12) and moderate-to-severe (GAP stage II/III; IPF, n = 13; non-IPF, n = 13) groups. In terms of clinical background, the only significant differences between the IPF and non-IPF groups were in the fraction of inspired oxygen, oxygen flow, and C-reactive protein levels (Table 1).

**Table 1.** Comparison of patients’ characteristics between the IPF and non-IPF groups

	IPF (n = 20)	Non-IPF (n = 25)	p value
Age at admission (years)	76 ± 5.8	75.3 ± 4.9	0.63
Gender (n/%)	Male, 13/65; female, 7/35	Male, 11/44; female, 14/56	0.16
Height (cm)	155.7 ± 10.3	150.6 ± 9.2	0.11
BMI (kg/m^2^)	21 ± 2.8	21.9 ± 3.2	0.22
Former smoker (n/%)	11/55	13/52	
Disease duration (years)	1.8 ± 1.4	2.1 ± 1.9	0.54
Hospital stay (days)	22.0 (16.0–26.8)	20.0 (14.5–24.5)	0.23
Causes of hospitalization (n/%)			
Scrutiny for diagnosis	3/15	5/20	0.72
Medication adjustment	7/35	11/44	0.55
Oxygen	8/40	8/32	0.76
Acute exacerbation	2/10	1/4	0.58
Diagnosis (n/%)	IPF, 20/100	NSIP, 2/8; CHP, 2/8; CTD-ILD, 8/32; sarcoidosis, 1/4; unclassified, 12/48	
GAP index (n/%)	I (7/35) II/III (13/65)	I (12/48) II/III (13/52)	0.41
Oxygen inhalation devices (n/%)	Room air, 2/10; nasal cannula, 18/90	Room air, 7/28; nasal cannula, 18/72	0.13
Oxygen flow (L/min)	3.0 (1.2–4.0)	2.0 (0.5–3.0)	<0.05
FiO_2_ (%)	32 (21–36)	28 (21–32)	<0.05
PaO_2_ (torr)	73 ± 6.6	74 ± 7.2	0.58
PaCO_2_ (torr)	40.8 ± 3.2	40.6 ± 3.1	0.85
%VC (% pred)	60.2 ± 13.7	67.1 ± 11.9	0.06
%DL_CO_ (% pred)	51.5 ± 12.1	54.7 ± 11.1	0.31
EF (%)	63.5 ± 5.8	65.4 ± 5.7	0.67
E/e′	7.6 ± 1.6	7.6 ± 1.4	0.95
TRPG (mmHg)	30.4 ± 8.7	29 ± 4.4	0.49
ALB (mg/dL)	3.7 ± 0.4	3.8 ± 0.3	0.70
CRP (mg/dL)	0.4 ± 0.4	1.1 ± 1.2	<0.01
KL-6 (U/mL)	1557.8 ± 988.2	1391.7 ± 1029.6	0.54
NT-proBNP (pg/mL)	138.5 ± 124.1	102.7 ± 88.7	0.24
Knee extension (N)	189.7 ± 48	190.6 ± 49.7	0.96

Values are presented as the mean ± standard deviation or as the median (1st quartile–3rd quartile).

IPF, idiopathic pulmonary fibrosis; BMI, body mass index; NSIP, nonspecific interstitial pneumonia; CHP, chronic hypersensitivity pneumonitis; CTD-ILD, connective tissue disease-associated interstitial lung disease; GAP index, gender–age–physiology index; FiO_2_, fraction of inspired oxygen; PaO_2_, partial pressure of arterial oxygen; PaCO_2_, partial pressure of arterial carbon dioxide; pred, predicted; %VC, %vital capacity; %DLco, %diffusing capacity for carbon monoxide; EF, ejection fraction; E/e’, ratio of early transmitral flow velocity to early diastolic velocity of the mitral annulus; TRPG, tricuspid regurgitation pressure gradient; ALB, albumin; CRP, C-reactive protein; KL-6, sialylated carbohydrate antigen KL-6; NT-proBNP, N-terminal pro-brain natriuretic peptide

### 6MWT assessment

In GAP stage I, patients with IPF had significantly lower values for the 6MWD (p < 0.05), minimum SpO_2_ level (p < 0.01), and HRR1 (p < 0.05) than did patients with non-IPF (Table 2). A decrease in FVC was observed in IPF compared with non-IPF in GAP stage I, but no difference was observed in GAP stage II/III (Table 3). By contrast, in GAP stage II/III, there were no significant differences in any 6MWT parameter between the IPF and non-IPF groups (Table 3).

**Table 2.** Comparison of 6MWT parameters between the IPF and non-IPF groups

	IPF (n = 20)	Non-IPF (n = 25)	p value		
			IPF vs. non-IPF	IPF at start vs. at end	Non–IPF at start vs. at end
SpO_2_ at start	96.3 ± 1.0	96.6 ± 1.2	0.27	<0.01	<0.01
SpO_2_ at end	80.2 ± 5.6	86.9 ± 5.0	<0.001		
HR at start	77.5 ± 9.1	75.1 ± 8.1	0.40	<0.01	<0.01
HR at end	120.0 ± 10.1	117.2 ± 10.0	0.46		
mBS at start	0 (0–0)	0 (0–0)	0.93	<0.01	<0.01
mBS at end	6 (5–7)	5 (4–6)	<0.05		

Values are presented as the mean ± standard deviation or as the median (1st quartile–3rd quartile). 6MWT, 6-minute walk test; IPF, idiopathic pulmonary fibrosis; SpO_2_, minimum percutaneous oxygen saturation; HR, heart rate; mBS: modified Borg scale score

**Table 3.** Comparison of the primary endpoints between patients with IPF and patients with non-IPF for each GAP stage

	IPF		Non-IPF		F value, p value		
					Main effect		Interaction
	GAP stage I	GAP stage II/III	GAP stage I	GAP stage II/III	GAP stage I IPF vs. non-IPF	GAP stage II/III IPF vs. non-IPF	
6MWD	301.3 ± 85.2	231.2 ± 107.1	410.1 ± 63.1	302.2 ± 150.3	F = 4.74, p < 0.05	F = 2.73, p = 0.11	F = 0.33, p = 0.57
SpO_2_ at end	83.4 ± 2.9	79.0 ± 6.6	90.5 ± 3.0	82.3 ± 4.1	F = 11.58, p < 0.01	F = 3.39, p = 0.07	F = 1.90, p = 0.18
HRR1	10.1 ± 4.9	9.1 ± 8.6	16.8 ± 6.1	14.4 ± 6.7	F = 4.09, p < 0.05	F = 2.67, p = 0.11	F = 0.09, p = 0.77
mBS	5.4 ± 1.0	6.1 ± 1.4	4.5 ± 1.1	5.7 ± 1.0	F = 2.73, p = 0.12	F = 0.55, p = 0.46	F = 0.59, p = 0.45
%FVC	79.2 ± 7.8	49.9 ± 12.5	91.8 ± 10.9	48.3 ± 13.3	F = 4.28, p < 0.05	F = 0.13, p = 0.72	F = 3.53, p = 0.07

Values are presented as the mean ± standard deviation. IPF, idiopathic pulmonary fibrosis; GAP, gender–age–physiology; 6 MWD, 6-minute walk distance; SpO_2_, minimum percutaneous oxygen saturation; HRR1, heart rate recovery at 1 minute after the 6 MWT; mBS, modified Borg scale score; %FVC, percent forced vital capacity

### PPF prevalence

With regard to the rate of PPF, in GAP stage I, there were more cases of patients with IPF (5/7, 71%) than patients with non-IPF (3/12, 25%). However, in GAP stages II and III, there were 11/13 (85%) cases of PPF among patients with IPF and 9/13 (69%) cases among patients with non-IPF. Among the cohort, 6 (30%) patients with IPF and 5 (20%) patients with non-IPF had diffusion disorders, all of which were in the severe GAP stage II/III (Table 4); thus, the diffusion impairment was significantly greater at GAP stage II/III than at GAP stage I for both the IPF and non-IPF groups, but there was no significant difference between the IPF and non-IPF groups.

**Table 4.** Comparison of diffusion impairments in patients with IPF and non-IPF at each GAP stage

	IPF				Non-IPF				p value			
n (%)	GAP stage I		GAP stage II/III		GAP stage I		GAP stage II/III		GAP stage I vs. II/III in IPF	GAP stage I vs. II/III in non-IPF	IPF vs. non-IPF in GAP I	IPF vs. non-IPF in GAP II/III
DLco 45% or more	7	(35)	7	(35)	12	(48)	8	(32)	<0.05	<0.05	—	0.44
Less than DLco 45%	0	(0)	6	(30)	0	(0)	5	(20)				

IPF, idiopathic pulmonary fibrosis; GAP, gender–age–physiology; %DLco, percent diffusing capacity for carbon monoxide

## Discussion

In this study, we performed a disease severity-based assessment of 6MWT parameters in patients with IPF and non-IPF, which, to the best of our knowledge, has not been similarly described in prior studies. In the case of moderate-to-severe disease, the GAP stage-related comparison indicated no significant differences in hypoxemia, endurance, or circulatory response, whereas in the case of mild disease, patients with IPF exhibited more pronounced hypoxemia, reduced endurance, and inadequate circulatory response compared to patients with non-IPF.

We observed no significant difference in dyspnea severity between patients with IPF and non-IPF. In contrast, FVC significantly differed between the 2 patient groups at GAP stage I, but not at GAP stage II or III. Previous studies have described that FVC reflects respiratory function at rest and does not necessarily correlate with dyspnea severity^18)^. Therefore, dyspnea might exhibit a different pattern from reduced FVC or advanced pulmonary fibrosis.

In GAP stage I (i.e., mild phase), the 6MWD, minimum SpO_2_ during the 6MWT, HRR1, and FVC were significantly lower in patients with IPF than in patients with non-IPF. IPF patients are more likely to develop advanced pulmonary fibrosis and lower FVC than non-IPF^19)^. Decreased FVC presents ventilation-perfusion inequality due to alveolar hypoventilation, which affects 6MWD reduction and SpO_2_ reduction during the 6MWT^20)^. Consequently, IPF patients may be more prone to reduced exercise capacity and hypoxemia, indicating a potential need to adjust oxygen dosage and walking distance even at GAP stage I. This finding suggests that oxygen administration may be considered in advance for patients with IPF at GAP stage I in anticipation of exertional hypoxemia during the 6MWT.

In GAP stage II/III (i.e., moderate-to-severe phase), no significant differences were found in 6MWT parameters between patients with IPF and patients with non-IPF. Previous studies have reported that exertional hypoxemia appears equally in IPF and non-IPF with PPF^7)^. In this study, 85% of the patients with IPF and 45% of those with non-IPF exhibited PPF, and most of these patients were considered to have GAP stage II/III. A previous study has indicated that patients with non-IPF presenting with PPF demonstrate pulmonary function impairment similar to that observed in patients with IPF^21)^. These findings suggest that the increased number of patients with non-IPF exhibiting PPF may contribute to the presence of exertional hypoxemia and reduced exercise tolerance. In cases of advanced respiratory disease, differences in exertional hypoxemia and 6MWD may be less pronounced due to the shared presence of pulmonary fibrosis and diffusion impairment. Unlike GAP stage I, hypoxemia and endurance loss are more likely to occur during the 6MWT in both patients with IPF and non-IPF, highlighting the necessity to consider oxygen administration and walking distance during the test.

In this study, a large number of patients in GAP stage II/III, both with IPF and non-IPF, exhibited reduced pulmonary diffusion capacity. A DLco value of 45% was used as the cutoff for pulmonary diffusion impairment; patients with a DLco below 45% are more likely to experience pulmonary hypertension on exertion, even in the absence of resting pulmonary hypertension^16)^. As the disease progresses, the pulmonary vascular bed and blood flow decrease, resulting in further diffusion impairment^22,23)^. Therefore, both IPF and non-IPF patients with diffusion impairment may be more prone to exertional hypoxemia and reduced endurance.

Additionally, many patients in GAP stage II/III were found to have decreased HRR1. Previous studies suggest that an HRR1 ≥13 bpm is considered normal, while an HRR1 ≤12 bpm is associated with a poor prognosis in ILD^24)^. Patients with ILD have an active cardiac compensatory mechanism for reduced lung function; however, prolonged compensation can overload the heart, leading to a suboptimal circulatory response^25,26)^. Furthermore, ILD patients with pulmonary hypertension demonstrate an even poorer circulatory response, with lower HRR1 values^27)^. These observations suggest that patients with non-IPF in GAP stage II/III may exhibit severe hypoxemia and reduced endurance, similar to patients with IPF.

Study limitations include the following. First, this is a single-center cross-sectional study and has limited generalizability. Therefore, a multicenter, cohort study may be required for generalization to other populations and to establish causal relationships for each 6MWT parameter by severity. Second, this study included only patients who were able to complete the 6MWT, and thus, it included many patients with mild to moderate disease. For severe cases with difficulty performing the 6MWT, it may be necessary to use an evaluation index other than 6MWT. Third, the diagnoses of IPF and non-IPF in this study were based on clinical diagnosis, and no surgical lung biopsy was performed. Therefore, we excluded cases in which it was difficult to classify as IPF or non-IPF. However, the accuracy of the clinical diagnosis is considered to be relatively high because the decision was made by a specialist in ILD and a diagnostic imaging physician. Lastly, we did not evaluate circulatory dynamics during the 6MWT in the present study. In the future, it will be necessary to measure maximal oxygen uptake and cardiac output during the 6MWT to quantitatively demonstrate changes in respiratory and circulatory dynamics. Considering the above study limitations, future perspectives are as follows. A multicenter cohort study including patients who have difficulty walking is needed for the generalization of the assessment of exertional hypoxemia in patients with ILD. Assessment of cardiokinetics will also help clarify whether the reason for difficulty in sustaining exercise is due to respiratory function or cardiokinetics.

## Conclusions

In mild disease, hypoxemia appeared more frequently in patients with IPF than in those with non-IPF, and this was accompanied by decreased endurance and a poor circulatory response. By contrast, there were no differences in hypoxemia, endurance, or circulatory response between non-IPF and IPF patients with moderate-to-severe disease. The significance of this study is to determine the degree of hypoxemia and endurance by severity in IPF and non-IPF patients, which could predict hypoxemia and endurance loss during the 6MWT and help manage risk.

## Acknowledgments

We would like to thank the patients who participated in this study and the rehabilitation staff of the International University of Health and Welfare, Ichikawa Hospital, for their cooperation in data collection. We would also like to thank the members of the Internal Research Laboratory, Graduate School of Health and Welfare, Saitama Prefectural University, for their frequent discussions regarding this study. We would also like to express our deepest gratitude to Professor Hiroshi Maruoka for his suggestions regarding interpretation of the results and discussions.

## Funding

Masashi Zenta is supported by a Grant-in-Aid for Scientific Research (Grant Number JP23K16580).

## Conflict of Interest

All authors declare that they have no conflict of interest.
